# Association between Western Dietary Patterns, Typical Food Groups, and Behavioral Health Disorders: An Updated Systematic Review and Meta-Analysis of Observational Studies

**DOI:** 10.3390/nu16010125

**Published:** 2023-12-29

**Authors:** Huang Zhang, Maiquan Li, Lan Mo, Jie Luo, Qingwu Shen, Wei Quan

**Affiliations:** 1 School of Food Science and Bioengineering, Henan University of Animal Husbandry and Economy, No. 6, Longzihu North Road, Zhengzhou 450046, China; 81870@hnuahe.edu.cn; 2 College of Food Science and Technology, Hunan Agricultural University, Changsha 410128, China; limaiquan@hunau.edu.cn (M.L.); molan@hunau.edu.cn (L.M.); luojie@hunau.edu.cn (J.L.)

**Keywords:** western dietary patterns, behavioral health disorders, red meat, refined grain, depression

## Abstract

Western dietary patterns (WDP) and typical food groups may play a major role in the risk of behavioral health disorders. Nevertheless, the relationships between WDP, common food categories, and mental health disorders lack consistency and remain incompletely understood in relation to potential mechanisms. Therefore, the objective of the present study was conducted to synthesize available evidence linking WDP and typical food groups to these outcomes. Web of Science, PubMed, EMBASE, and MEDLINE were searched up to August 2023. Random effect meta-analyses were performed to obtain pooled odds ratio and the relative risk for the prevalence of outcomes and the incidence of outcomes, respectively. A total of 54 articles were included. WDP was associated with increased risk of both depression (1.19; 95% CI: 1.06–1.32) and depressive symptoms (1.20; 95% CI: 1.08–1.34). Except for high-fat dairy products, food groups are associated with an increased risk of anxiety, depression, and depressive symptoms. This review presents evidence to further understand the relationship between WDP, typical food groups, and the incidence of behavioral health disorders, and more randomized controlled trials and cohort studies are urgently required to confirm these findings and elucidate potential mechanisms.

## 1. Introduction

Behavioral health encompasses a wide range of mental and emotional well-being behaviors and conditions. It covers various aspects, including the ability to cope with everyday life challenges, as well as the presence of mental health disorders like depression, anxiety, and other psychiatric conditions [1,2]. According to the Global Burden of Disease study, it was discovered that behavioral disorders were responsible for 22.6% of the total years spent living with disability [1,3]. According to the World Health Organization, depression is a prevalent behavioral health disorder in the general population that typically manifests with symptoms of sadness, fatigue, and loss of interest in daily activities, and thus is regarded as the primary reason for disability and a major cause for disease burden worldwide [4,5,6]. The current treatments for depression are associated with problems like expensive medication, negative side effects, and inadequate effectiveness. Thus, it is crucial to identify modifiable dietary factors to prevent behavioral health disorders.

Generally, behavioral health disorders are complicated diseases caused by interaction of genetic and environmental factors [4]. Although the pathophysiology of behavioral health disorders remains vague, existing evidence suggests that modifiable and environmental factors such as diet and physical activity contribute to the onset of the disease [4,7,8]. Over the past decade, epidemiological evidence on the relationship between dietary patterns and mental health has been increasing [5,9]. A healthy diet such as the Mediterranean diet, etc., has been associated with better behavioral health [10,11]. In contrast, an “unhealthy diet” such as the Dietary Inflammatory Index leads to an inflammatory dietary pattern due to the role of inflammation in the pathogenesis of depression and eventually a higher risk of behavioral health disorders [12]. The Western dietary pattern (WDP) is a proinflammatory diet mainly characterized by a high consumption of red and/or processed meat, refined grains, sugar-sweetened beverage (SSB), high-fat dairy (HFD) products, butter, potatoes and high-fat gravy, and low intakes of fruits and vegetables [13]. We speculate that long-term adherence to WDP may be an important factor contributing to increased risk of mental health disorders. And some studies have evaluated the association between WDP and behavioral health disorders. For example, a stronger commitment to following the WDP was linked to higher scores on the Child Behavior Checklist, which led to poorer mental health outcomes [14]. However, the findings in the previous literature are not consistent possibly due to a methodological difference. Additionally, due to the potential impact of foods on health and well-being, there has been significant curiosity regarding the effects of specific food groups on mood and brain function [3,15]. However, as far as we know, there have been limited studies examining the potential connection between particular food groups and the likelihood of behavioral health disorders [9,16,17].

The aim of present study was to synthesize all available observational studies that evaluated the association between WDP, consumption of typical WDP food groups (refer to the definition of WDP including, red or processed meat, refined grains, sugar-sweetened beverage, high-fat dairy products, fast food), and behavioral health disorders (i.e., anxiety, depression, and depressive symptoms).

## 2. Materials and Methods

The present systematic review and meta-analysis was registered (PROSPERO ID: CRD42023470751). and conducted in line with the PRISMA [18] (Supplemental Table S1) and MOOSE [19] (Supplemental Table S2). 

### 2.1. Search Strategy

The PubMed, MEDLINE, Web of Science, and EMBASE databases were searched up to August 2023. Each database included the use of “western dies” OR “western dietary patterns” OR “red meat” OR “refined grain” OR “fast food” OR “high fat dairy” OR “soft drink” OR “sweet drink” OR “sweet beverage” OR “sugar-sweetened drink” OR “carbonated beverage” OR “carbonated drink” AND “mental disorder” OR “mental health” OR “depression” OR “depressive disorder” OR “depressive symptoms” OR “anxiety” as MeSH and keywords. Only articles published in English were included, and there were no restrictions or filters in the search. We conducted a manual search in the reference lists of all retrieved literature for additional relevant studies. The literature search was conducted by two investigators separately. In case of data duplication across multiple studies, we prioritized including the study with the most extensive data. When the two investigators disagreed on the eligibility of an article, they reached a consensus.

### 2.2. Inclusion Criteria

The article titles and abstracts were first reviewed by two investigators separately, who then evaluated the full text using following inclusion criteria: (a) observational studies; (b) WDP and typical WDP food groups including red meat, HFD products, refined grain, SSB, and fast food as the exposure; (c) the outcome of interest was depression, anxiety, depressive symptoms, or psychological distress, and the definition was based on physicians’ diagnosis or professional questionnaires; and (d) available multivariable adjusted hazard ratio or OR and CI of the main outcome for the highest versus the lowest. If an article included cross-sectional results as well as prospective results, or if the results were reported for both soft drinks and other types of sweetened beverages, it was categorized as two studies.

### 2.3. Exclusion Criteria

Studies were excluded if they (a) focused on the effects of single nutrients or foods; (b) examined other psychiatric diseases as the main outcome; (c) had patients as sample subjects; (d) narrative articles that do not include primary data; or (e) were published in a language other than English.

### 2.4. Data Extraction and Methodological Quality Assessment

The data extraction and quality assessment of the included studies were carried out separately by two investigators. Any disagreements were resolved by consensus or consultation with a third investigator. To complete our dataset for studies with incomplete data, e-mails were sent to the corresponding authors of these studies.

The quality of the studies included in the systematic review was assessed using the NOS Scale, which assesses the methodological quality based on the quality of the original studies included. The NOS Scale is composed of three main components which include the cohort selection process (4 points), accounting for known confounding factors (2 points), and the diagnostic approach and criteria for exposure or outcome (3 points). A high NOS score (>6) represented better methodological quality.

### 2.5. Sensitivity Analysis and Subgroup Analysis

To evaluate the durability of the findings, a sensitivity analysis was conducted based on the quality evaluation outcomes. At each stage, an exclusion of a specific study was executed to ascertain the level of impact caused by an individual study or a cluster of studies on our results. Prespecified subgroup analyses were conducted if there was significant heterogeneity, considering the quality score of the study (quality score < 7 vs. quality score ≥ 7), gender of participants (female and male), and location of studies (western and eastern countries).

### 2.6. Statistical Analysis

Meta-analysis was performed to estimate the association between unhealthy dietary patterns and the risk of mental disorders. A random effects model was employed in the study to determine the combined odds ratios (OR) and corresponding 95% confidence intervals (95% CI). This calculation was based on comparing the highest intake to the lowest intake of each dietary pattern. Hazard ratios (HR) were considered equivalent to odds ratios (OR). In a sensitivity analysis, these studies were excluded to assess the impact of the inclusion of studies that reported HR on the overall result.

Random effects models were used to detect potential heterogeneity between diet types and study design. A heterogeneity test was conducted for each outcome using Cochran’s Q test and *I*^2^ statistics. A significance threshold of less than 0.1 was used for the *p*-values of the Q statistic to determine statistical significance. Regarding the *I*^2^ statistic, *I*^2^ scores below 25% indicated low levels of heterogeneity, while scores of 50% or higher indicated the presence of between-study heterogeneity. Furthermore, Egger’s test and visual inspection of a funnel plot for all meta-analyses were performed to assess potential publication bias. Statistical analyses in this study were conducted with STATA 14.0 (STATA Corp, College Station, TX, USA) and Review Manager 5.3 (The Cochrane Collaboration, Copenhagen, Denmark). Statistical significance was indicated by *p*-values below 0.05.

## 3. Results

### 3.1. Search Results and Study Characteristics

**Figure 1:**
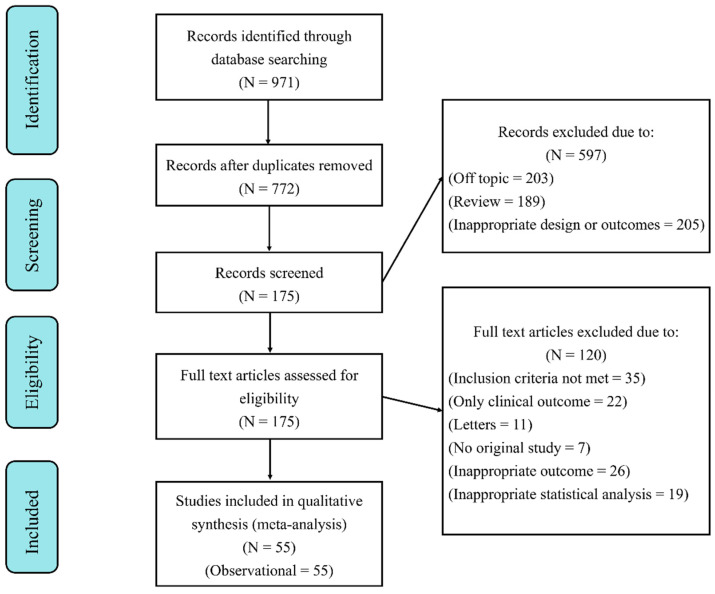
Flow diagram of the literature selection procedure.

A total of 357 articles were identified from PubMed, 425 articles from Web of Science, and 188 articles from MEDLINE (Figure 1). After removing duplicates, there were still a total of 772 articles remaining. Out of these, 597 articles were excluded after evaluating their titles or abstracts. From the initial 175 articles that qualified for a thorough full text assessment, 120 articles were eliminated for various reasons. Finally, 54 articles published between 2005 and 2023 were eligible for systematic review and meta-analysis.

### 3.2. Characteristics of Included Studies

Of the 54 included articles, 16 reported the association between WDP and behavioral health disorders, the other 38 focused on the typical food groups from WDP. The characteristics from observational studies assessing the effects of WDP and typical food groups on behavioral health disorder outcomes are shown in Table 1 and Table 2, respectively.

Twenty-five studies were conducted in Eastern countries (China, Iran, Korea, and Japan), while 29 studies were conducted in western countries (France, the United Kingdom, Australia, Greece, and Norway). The sample size ranged from 546 to 53,637, with a total number of more than 1 million individuals. The length of follow-up of the prospective cohort studies ranged from 1 to 17 years. Validated semiquantitative food frequency questionnaires were used to collected data on dietary intake from most studies: 16 studies focused on WDP, 8 on fast food, 17 on SSB, 15 on red meat, 3 on refined grain, and 3 on HFD products. Regarding the outcome of behavioral health disorders, 13 studies examined the effects of WDP and typical food group in terms of anxiety; 40 studies focused on the relationship between WDP, typical food group, and depression; and 16 articles evaluated depressive symptoms. In nine studies, behavioral health disorders were assessed by clinical physician diagnosis. However, the vast majority of the identified studies examined behavioral health disorders using self-reported depression scales and questionnaires. In most studies, CES-D, PHQ-9, and HADS were used. To investigate the relationship between WDP, typical food group, and behavioral health disorders, the GSHS, SDS, the Edinburgh Postpartum Depression Scale, the Depression Self-rating Scale for Children, the Children’s Depression Inventory, depression, anxiety and stress scale (DASS 21 items), and Beck Depression Inventory were used in some studies. Moreover, the quality assessment based on the NOS score showed that 13 articles were ranked as high quality, 3 as low quality, and 39 were moderate quality.

### 3.3. Western Dietary Pattern and Behavioral Health Disorders

**Figure 2:**
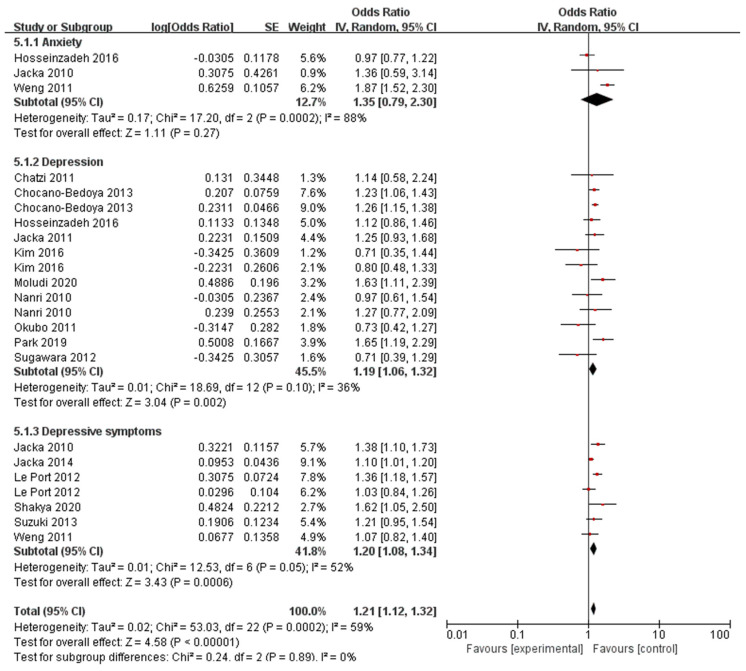
Meta-analysis of adhere to Western dietary patterns and the maximum-adjusted risk ratio of behavioral health disorders using the random effects model. CI, confidence interval [13,20,21,22,23,24,25,26,27,28,29,30,31,32,33,34].

A total of 16 articles reported the results of adherence to WDP and the incidence of behavioral health disorder. Out of these, three studies specifically examined anxiety, while thirteen studies were centered around depression, and seven studies explored depressive symptoms. Figure 2 shows the forest plot for the risk of three kind of behavioral health disorder outcomes in the highest compared with the lowest category of WDP. WDP was associated with an increased risk of depression (pooled OR = 1.19; 95% CI: 1.06–1.32) without significant heterogeneity (*I*^2^ = 36%, *p* = 0.10) and depressive symptoms (pooled OR = 1.20; 95% CI: 1.08–1.34) but with significant heterogeneity (*I*^2^ = 52%, *p* = 0.05). Moreover, WDP was not significantly correlated to the risk of anxiety (pooled OR = 1.35; 95% CI: 0.79–2.30), but there was still significant heterogeneity (*I*^2^ = 88%, *p* = 0.0002). Therefore, the random effects model was used to evaluate the impact, along with conducting additional subgroup analysis.

### 3.4. Fast Foods and Behavioral Health Disorders

**Figure 3:**
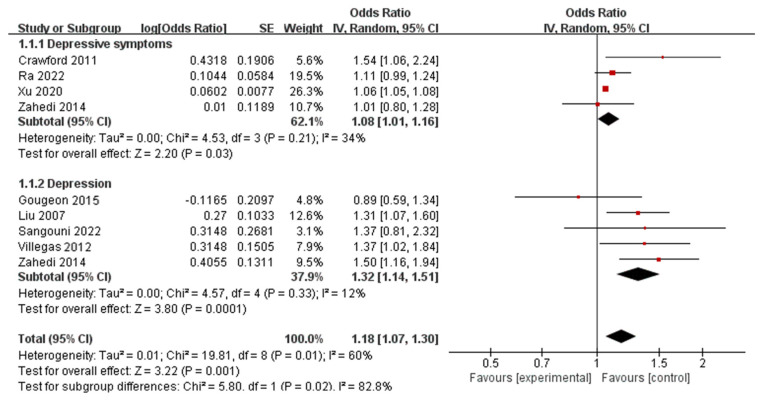
Meta-analysis of fast-food intakes and the maximum-adjusted risk ratio of behavioral health disorders using the random effects model. CI, confidence interval [16,35,36,37,38,39,40,49].

Earlier cohort studies involving adolescents have suggested that a high intake of fast food, which includes hotdogs, hamburgers, cheeseburgers, fried chicken, and pizza, is linked to an increased likelihood of experiencing behavioral problems and mental distress, such as anxiety, feelings of dizziness, and a sense of worthlessness. Our results revealed that four studies investigated the association between fast foods and five studies assessed the same with depression and depressive symptoms. The highest intake of fast food was significantly associated with behavioral health disorders, compared with the lowest category (for depressive symptoms, pooled OR 1.08, 95% CI: 1.01–1.16; for depression, pooled OR 1.32, 95% CI: 1.14–1.51) (Figure 3). No significant heterogeneity was noted (for depressive symptoms, *I*^2^ = 34%, *p* = 0.21; for depression, *I*^2^ = 12%, *p* = 0.33). The stability of the current results is confirmed as no significant changes were observed in the pooled odds ratios (ORs) and 95% confidence intervals (CIs) when any individual study was excluded during sensitivity analyses.

### 3.5. Red Meat and Behavioral Health Disorders

**Figure 4:**
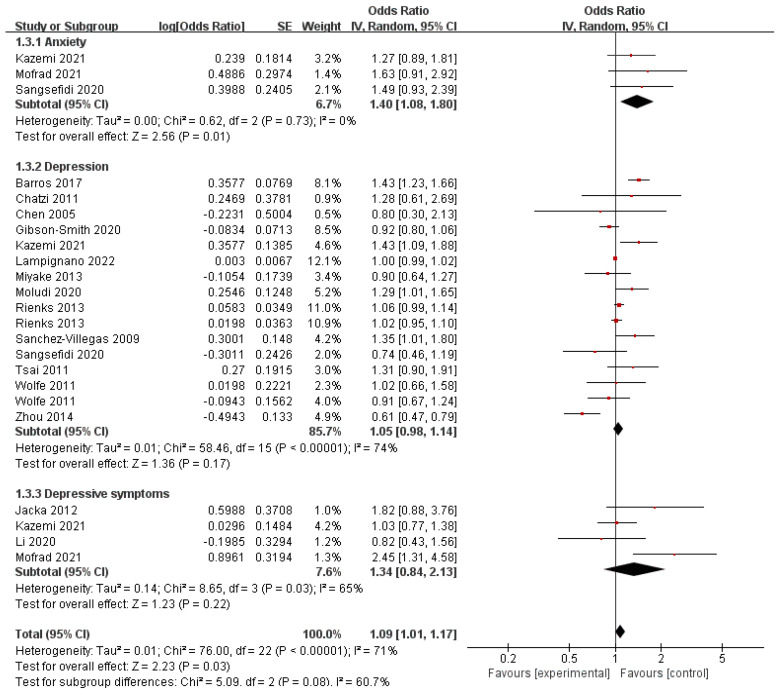
Meta-analysis of red-mesat intakes and the maximum-adjusted risk ratio of behavioral health disorders using the random effects model. CI, confidence interval [2,7,8,17,26,31,51,52,53,54,55,56,57,58,59,60].

Red meat, deemed as the most controversial food in the history of nutrition, plays a significant role in WDP. Red or processed meat consumption has been linked to elevated levels of proinflammatory cytokines and the potential development of behavioral health disorders. The connection between depression and meat consumption was investigated in a meta-analysis that included two case-control studies, three cohort studies, and three cross-sectional studies. The analysis revealed that there was no notable correlation found between the consumption of red meat and a heightened susceptibility to depression. However, certain cohort studies did report that meat consumption was linked to a 13% higher risk of depression. In the present meta-analysis, 23 studies reported the results of red-meat intakes and the outcome of behavioral health disorders (Figure 4). The pooled OR (1.40, 95% CI: 1.08–1.80) revealed that the highest versus the lowest consumption of red meat was significantly associated with an increased risk of anxiety, with non-significant evidence of heterogeneity (*I*^2^ = 0%, *p* = 0.73). Moreover, red-meat intake was not significantly associated with the incidence of depression (pooled OR 1.05, 95% CI: 0.98–1.14) and depressive symptoms (pooled OR 1.34, 95% CI: 0.84–2.13). The *I*^2^ value for heterogeneity was 74% and 65%, respectively, indicating substantial heterogeneity, as reflected by *p* < 0.01 for homogeneity.

### 3.6. Refined Grain and Behavioral Health Disorders

**Figure 5:**
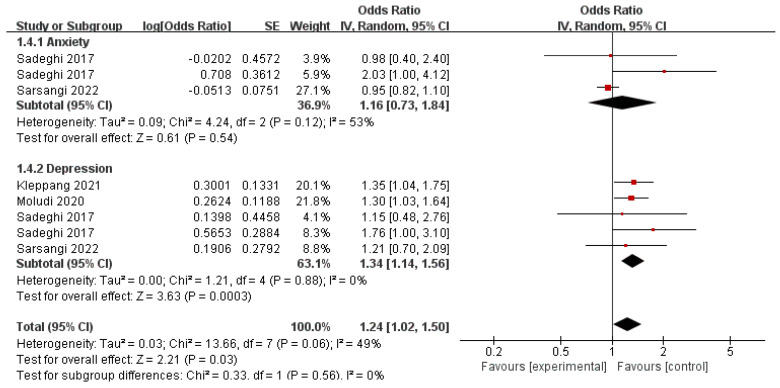
Meta-analysis of refined grain and the maximum-adjusted risk ratio of behavioral health disorders using the random effects model. CI, confidence interval [26,62,63,64].

A diet rich in red and processed meats, as well as refined sugar, but lacking in plant-based foods, could potentially impact mental disorders. Previous research has predominantly centered around the connection between depression and anxiety with the consumption of refined grains and foods with a high glycemic index (GI). In the present meta-analysis, we included eight studies to investigate the relationship between the consumption of refined grains and behavioral health disorders. Figure 5 shows a significantly increased risk of depression in the highest category of refined grain intakes (pooled OR = 1.34, 95% CI: 1.14–1.56; *p* < 0.0001), with non-significant evidence of heterogeneity (*I*^2^ = 0%, *p* = 0.88). In addition, refined grain intake was not significantly correlated to the risk of anxiety (pooled OR = 1.16, 95% CI: 0.73–1.84; *p* = 0.54). A random effects model was used to assess the included data and showed an apparent heterogeneity in all studies (*p* = 0.12; *I*^2^ = 53%).

### 3.7. Sugar-Sweetened Beverage and Behavioral Health Disorders

**Figure 6:**
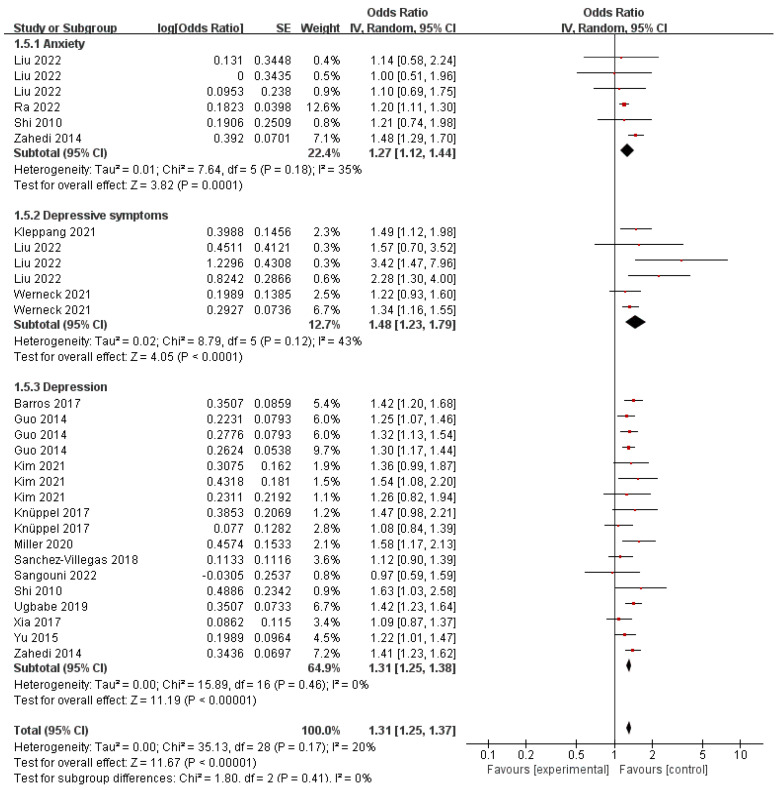
Meta-analysis of sugar-sweetened beverage and the maximum-adjusted risk ratio of behavioral health disorders using the random effects model. CI, confidence interval [2,6,15,16,39,41,42,43,44,45,46,47,48,49,50,62].

SSBs are rich in sugar and are being recognized as a significant dietary element that impacts mental well-being. Multiple studies have been carried out to establish the connection between SSBs and various health consequences: in the present meta-analysis, 6 studies, between SSBs intake and risk of anxiety; 6 on SSB intake; and 17 on the risk of depressive symptoms and depression. SSB intake was positively correlated to the risk of anxiety (pooled OR 1.27, 95% CI: 1.12–1.44), depressive symptoms (pooled OR 1.48, 95% CI: 1.23–1.79), and depression (pooled OR 1.31, 95% CI: 1.25–1.38) (Figure 6). Meanwhile, no significant heterogeneity was reported (*I*^2^ = 35%, *p* = 0.18 for anxiety; *I*^2^ = 43%, *p* = 0.12 for depressive symptoms; *I*^2^ = 0%, *p* = 0.46 for depression).

### 3.8. High-Fat Dairy and Behavioral Health Disorders

**Figure 7:**
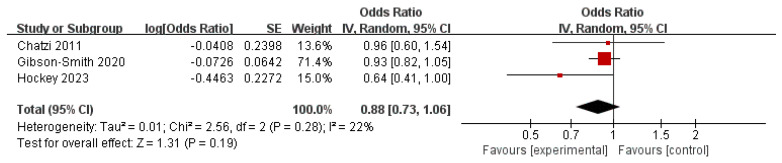
Meta-analysis of high-fat dairy intakes and the maximum-adjusted risk ratio of behavioral health disorders using the random effects model. CI, confidence interval [7,31,65].

Evidence is lacking on the association between HFD products and behavioral health disorders. Currently, due to the limited inclusion of studies, we identified only three studies that reported the results of HFD and incidence of depression (Figure 7), with the pooled OR of 0.88 (95% CI: 0.73–1.06) for the highest versus the lowest HFD intakes in a random effects model and non-significant evidence of heterogeneity (*I*^2^ = 22%, *p* = 0.28).

### 3.9. Publication Bias and Sensitivity Analysis

Supplemental Figures S1–S6 exhibit the contour-enhanced funnel plots representing the four primary dietary scores. A visual examination of the plots suggests a lack of publication bias. The estimates derived from the studies included were evenly spread out around the overall estimate for each dietary index. Furthermore, studies with both significant and non-significant estimates were encompassed in the analysis. In the sensitivity analysis, each study was sequentially excluded, yet did not show significant changes in the pooled RRs, indicating the robustness of the results.

### 3.10. Subgroup Analysis

To provide more details about the considerable variation in the study, subgroup analyses were conducted. These analyses divided the studies according to their locations and the number and gender of participants. Table 3 shows the results of the subgroup analyses stratified according to the aforementioned factors (raw data were shown in Figures S7–S11). There were significant changes in the relationship between WDP, red meat, and behavioral health disorder outcomes. In detail, subgroup analysis by the location of studies showed that in Eastern countries, WPD (pooled OR 1.48, 95% CI: 1.12–1.96), red meat (pooled OR 1.41, 95% CI: 1.24–1.61), and depression were significantly associated, but none among Western countries. Given that the best evidence on this association came from Eastern countries, the positive association of WDP and red meat with behavioral health disorders should be robust. When stratified by gender of participants, the association between WDP and depressive symptoms was stronger in men (pooled OR 1.36, 95% CI: 1.25–1.47), while there was no statistically significant difference in women. When stratified by the number of participants, red meat was significantly associated with an increased incidence of depression in studies with more than 10,000 participants (pooled OR 1.41, 95% CI: 1.24–1.61). Before definitively concluding, it is necessary to conduct additional prospective longitudinal studies in order to clarify the gender-specific inflammatory potential in the relationship between diet and depression.

## 4. Discussion

In the present study, the association between WDP, typical WDP food groups, and behavioral health disorders has been identified based on updated evidence. A total of 54 studies were identified and included in the present study, in which five kinds of typical WDP food group became apparent: fast food, red meat, refined grain, HFD products, and SSB. Results indicated that adherence to WDP was positively associated with the risk of depression and depressive symptoms, particularly in men and in individuals in Eastern countries. In addition, our findings further confirmed that typical WDP food groups, except for HFD, were significantly associated with a great variety of behavioral health disorders outcomes. Although in the current study, we acquired combined ORs that were slightly elevated compared to those reported in previous meta-analyses, but it might related to the inclusion of additional studies resulting in increased numbers [66]. Overall, our findings are in line with evidence from some previously published reference. For instance, although Sugawara et al. indicated no statistically significant association between the WDP and depression [20]. The investigation conducted by Nucci and colleagues revealed a link between the consumption of red and processed meat and the likelihood of experiencing depression [67].

Although the underlying mechanisms are not sufficiently understood, but refer to previous reported studies, there are several possible explanations or hypotheses for this adverse effect of the WDP, and typical WDP food groups on the risk of behavioral health disorders. Firstly, it has been found that adhering to the WDP, and typical WDP food groups can result in the ongoing activation of the immune system, leading to an increase in the production of ROS. These ROS are known to be associated with low-grade proinflammatory responses, ultimately leading to chronic low-grade inflammation. The increased levels of ROS and inflammatory markers are directly associated with apoptosis and cerebral atrophy, particularly in the hippocampus, which finally increases the risk of developing behavioral health disorders [21,23,68]. Therefore, the most important and directly mechanisms related to WDP and behavioral health disorders are the chronic subclinical inflammation and corresponding increased oxidative stress caused by WDP [69,70]. Secondly, another possible pathway explains the indirect impact of WDP and typical food groups on behavioral health disorders. Since proinflammatory foods are often closely associated with the risk of chronic diseases such as atherosclerosis, and diabetes. While those chronic diseases are also related to depression, cerebrovascular diseases, and brain atrophy [6,16,58]. Additionally, the detrimental impact of the Western-style eating habit may arise due to excessive intake of sugar found in candies and carbonated beverages [41,45]. This could be due to several factors, such as an increase in the reactivity of the hypothalamic–pituitary–adrenal (HPA) axis, which disrupts the body’s ability to respond to stress. Another possible explanation is that excessive sugar intake leads to obesity or insulin resistance, which in turn causes chronic low-grade inflammation and non-habituation of the HPA axis. These effects may contribute to the development of depression [71].

In addition, the WDP food group are usually subjected to various heat processing methods, resulting in the loss of valuable components (i.e., amino acids derived from proteins, vitamins, fiber, and minerals). These components have an important impact on mental health, such as the conversion of tryptophan into serotonin, which is a key regulatory factor in emotions and can produce similar anti-depressive effects. Tyrosine also affects emotions as a precursor of dopamine. Furthermore, research has shown that folate deficiency may lead to an increase in homocysteine concentration and a decrease in S-adenosylmethionine availability, which plays a key role in the pathophysiology of depression [72]. Furthermore, previous research has suggested a correlation between increased antioxidant levels and decreased oxidative stress, which is believed to have positive effects on mental well-being [69,70]. These antioxidants found in fruits and vegetables, such as vitamins and phenolic compounds, may have a protective effect against depression. However, in WDP, the intake of these foods is insufficient and these antioxidants are easily degraded and can trigger other reactions during the cooking processes, resulting in a decrease in their antioxidant activity [73].

Finally, WDP food groups are highly processed, and apart from losing valuable components, some low-quality ingredients are added, and some harmful products are generated during thermal processing, which are involved in the pathogenesis of depression. For example, despite the large amounts of protein, minerals, and B vitamins that are essential for the proper functioning of neurotransmitters and improvement of mental health, red meat also contains a relevant source of cholesterol, saturated fatty acids, and arachidonic acids that could elevate levels of proinflammatory cytokines and further disrupt neurotransmitter metabolism pathways, reduce plasma tryptophan levels, and prevent the expression of brain-derived neurotrophic factor (BDNF) [54,61,67]. BNFD is a peptide critical for optimal neuronal function, which decreases in depression [74]. Given that WDP food groups are primarily subjected to high-temperature methods like frying and baking, it is possible that the association between WDP and mental health risk is linked to the formation of detrimental substances during the Maillard reaction. These substances include advanced glycation end products (AGEs), heterocyclic amines, and acrylamide [75,76]. Since, our previous studies have confirmed that those harmful compounds could be promoting oxidative stress and neuroinflammation through the blood–brain barrier, which consequently leads to neurocognitive deficiencies that closely resemble those documented in individuals experiencing major depressive disorder [77,78]. Currently, these associations lack strength and persuasion, and additional evidence is necessary to confirm the previously mentioned potential ways in which hazardous products in heat-processed meat products may contribute to an elevated risk of cognitive impairment.

The present meta-analysis is strong due to the inclusion of a significant number of studies and participants, as well as the satisfactory methodologic quality of the analyzed studies. Sufficient statistical power was used to derive more generalizable and definitive conclusions. However, there were some limitations. First, measurement errors were inevitable due to the nature of the meta-analysis, which was based on observational studies. Second, the evaluation of food consumption primarily relied on self-reported habits, which are prone to potential recall errors. In addition, the analysis included studies that used different methodological approaches, including various measurements for depression scales. The majority of these studies utilized questionnaires, particularly the CES-D, although with different versions, while some questionnaires were exclusively used in one specific study. Only a minority of studies examined clinical depression, assessed by clinical interview or self-reported physician diagnosis. As a result, there was significant heterogeneity observed among the studies. Lastly, as the present meta-analysis was based on observational studies, potential confounders could not be ruled out and thus affect the relationship between WDP, typical food group, and risk of behavioral health disorders. Therefore, more studies are needed to obtain more credible research evidence.

## 5. Conclusions

The present systematic review and meta-analysis of 54 observational studies provides a comprehensive overview and critical evidence of the currently available studies focusing on the relationship between adhere to WDP, consumption of typical WDP food groups, and behavioral health disorders. Our findings confirmed that the current trends in which a high consumption of WDP is associated with a higher risk of behavioral health disorder outcomes. Except for HFD products, typical WDP food groups are associated with an increased risk of anxiety, depression, and depressive symptoms. To elucidate whether true causal associations exist between WDP, consumption of typical WDP food groups, and behavioral health disorders, further research is urgently needed to elucidate the potential mechanisms.

## Figures and Tables

**Table 1:** Study design details and population characteristics from observational studies assessing the effects of western dietary patterns on behavioral health disorder outcomes.

No.	Authors, Year, Country of Study	N = Subjects (Case)	Age; Year	Outcome Assessment (Diagnosis Criteria)			Quality Score
				Type	Tool	Cut-Off	
1	Sugawara, 2012, [20] Japan	791 (31)	22–86	Depression	CES-D	≥16	5
2	Park, 2019, [21] Korea	338 (448)	40–69	Depression	BDI	≥16	6
3	Jacka, 2014, [22] Australia	3663 (343)	20–64	Depressive symptoms	GDS	≥6	6
4	Chocano-Bedoya, 2013, [23] U.S	50,605 (3002)	50–77	Depression	Clinical diagnosis	-	8
5	Shakya, 2020, [24] Australia	1743 (86)	>24	Depressive symptoms	CES-D	≥16	6
6	Kim, 2016, [25] U.S	4180 (836)	20–79	Depression	PHQ-9	≥10	5
7	Moludi, 2020, [26] Iran	4630 (273)	25–65	Depression	Clinical diagnosis	-	6
8	Jacka, 2010, [13] Australia	1046 (60)	20–93	Depressive symptoms/Anxiety	GHQ-12/SCID-I/NP	-	5
9	Nanri, 2010, [27] Japan	521 (56)	21–67	Depression	CES-D	≥16	5
10	Jacka, 2011, [28] Norway	3254 (281)	-	Depression/Anxiety	HADS	≥8	6
11	Le Port, 2012, [29] France	9272 (630)	35–50	Depressive symptoms	CES-D	≥17 (M) ≥23 (F)	7
12	Okubo, 2011, [30] Japan	865 (121)	29.9	Depression	EPDS	≥9	4
13	Chatzi, 2011, [31] Greece	529 (176)	-	Depression	EPDS	≥13	4
14	Hosseinzadeh, 2016, [32] Iran	3846 (525)	20–55	Depression/Distress/Anxiety	HADS	≥8	6
15	Weng, 2012, [33] China	5003 (560)	11–16	Depressive symptoms/Anxiety	DSRS/SCARED	≥15	5
16	Suzuki, 2013, [34] Japan	2266 (167)	21–65	Depressive symptoms	K6 scale	≥9	5

Abbreviations of diagnosis criteria: EPDS, Edinburg Postpartum Depression Scale; BDI, Beck Depression Inventory; CES-D, Center for Epidemiologic Studies Depression Scale; K6, Kessler Psychological Distress Scale; PHQ-9, Patient Health Questionnaire 9-item depression module; HADS, Hospital Anxiety and Depression Scale; GDS, Goldberg Depression scale, GHQ-12, General Health Questionnaire 12 items; SCID-I/NP, Structured Clinical Interview for DSM-IV-TR Research Version; DSRS, the Depression Self-rating Scale for Children; SCARED, the Chinese version of the Screen Scale for Child Anxiety Related Emotional Disorders.

**Table 2:** Study design details and population characteristics from observational studies assessing the effects of typical Western dietary pattern food groups on behavioral health disorder outcomes.

No.	Authors, Year, Country of Study	N = Subjects (Case)	Age; Year	Outcome Assessment (Diagnosis Criteria)			Type	Quality Score
				Type	Tool	Cut-off		
1	Crawford, 2011, [35] U.S	626 (155)	45–54	Depressive symptoms	CES-D	≥16	FFP	4
2	Villegas, 2012, [36] Spain	10,374 (118)	-	Depression	SCID-I	-	FFP	7
3	Liu, 2007, [37] Norway	2579 (368)	-	Depression	CES-D	≥16	FFP	6
4	Gougeon, 2015, [38] Canada	1358 (170)	67–84	Depression	Geriatric Depression Scale	≥11	FFP	5
5	Ra, 2022, [39] Korea	24,006 (19,806)	<18	Depressive symptoms/Anxiety	Clinical diagnosis	-	SSB/FFP	6
6	Xu, 2020, [40] China	14,500 (4217)	<20	Depressive symptoms	CDI	≥20	SSB/FFP	7
7	Liu, 2022, [41] China	1311 (183)	7–17	Depressive symptoms/Anxiety	CDI/SASC	≥20	SSB	7
8	Kim, 2021, [6] Korea	5465 (739)	>20	Depression	PHQ-9	≥5	SSB	5
9	Miller, 2020, [42] Australia	3430 (387)	-	Depression	Clinical diagnosis	-	SSB	5
10	Ugbabe, 2019, [43] U.S	53,637 (10,597)	>18	Depression	Clinical diagnosis	-	SSB	7
11	Werneck, 2021, [44] Spain	25,920 (3715)	42.9	Depressive symptoms	PHQ-9	≥10	SSB	7
12	Guo, 2014, [15] U.S	10,524 (653)	61.5	Depression	Clinical diagnosis	-	SSB	5
13	Sanchez-Villegas, 2018, [45] Spain	15,546 (769)	-	Depression	Clinical diagnosis	-	SSB	5
14	Knüppel, 2017, [46] UK	9895 (1229)	35–55	Depression	CES-D	≥16	SSB	6
15	Yu, 2015, [47] China	3667 (2565)	42.5	Depression	SDS	≥40	SSB	5
16	Barros, 2017, [2] Brazil	49,025 (5144)	37	Depression	PHQ-9	≥20	SSB/Red meat	7
17	Xia, 2017, [48] China	2702 (1351)	46.2	Depression	SDS	≥45	SSB	6
18	Zahedi, 2014, [49] Iran	13,486 (2794)	-	Depression/Anxiety	GSHS	-	SSB/FFP	7
19	Shi, 2010, [50] Australia	4741 (326)	>16	Depression/Anxiety	Clinical diagnosis/K10	≥22	SSB	6
20	Sangsefidi, 2020, [51] Iran	9965 (1651)	20–69	Depression/Anxiety	DASS 21 items	≥10	Red meat	7
21	Gibson-Smith, 2020, [7] Netherland	1634 (414)	18–65	Depression	IDS/BAI/FEAR	-	Red meat/HFD	5
22	Rienks, 2013, [52] Australia	8369 (1742)	50–55	Depression	CES-D	≥10	Red meat	7
23	Tsai, 2011, [53] Taiwan	1609 (203)	>65	Depression	CES-D	≥10	Red meat	6
24	Wolfe, 2011, [17] U.S	1962 (223)	25–74	Depression	CES-D	≥16	Red meat	6
25	Kazemi, 2021, [54] Iran	3362 (962)	18–55	Depression	HADS/GHQ	≥4	Red meat	6
26	Mofrad, 2021, [55] Iran	482 (128)	20–50	Depressive symptoms	DASS 21 items	≥10	Red meat	5
27	Chen, 2005, [56] China	1600 (142)	>60	Depression	GMS	-	Red meat	5
28	Sanchez-Villegas, 2009, [57] Spain	10,094 (480)	37.2	Depression	Clinical diagnosis	-	Red meat	6
29	Miyake, 2013, [58] Japan	1745	31.2	Depression	CES-D	≥16	Red meat	5
30	Zhou, 2014, [8] China	11,473	>65	Depression	PHQ-9	≥10	Red meat	5
31	Li, 2020, [59] U.S	17,845 (1647)	18–65	Depressive symptoms	PHQ-9	≥10	Red meat	7
32	Jacka, 2012, [60] Australia	1046 (60)	20–93	Depressive symptoms	SCID-I/NP	-	Red meat	5
33	Lampignano, 2022, [61] Italy	546	-	Depression	DSM-IV-TR	-	Red meat	5
34	Kleppang, 2021, [62] Norway	2230	-	Depressive symptoms	CONOR-MHI	≥2.15	SSB	5
35	Sadeghi, 2017, [63] Iran	1398	18–55	Depression/Anxiety	HADS	-	Refined grain	5
36	Sarsangi, 2022, [64] Iran	7574 (1333)	20–70	Depression/Anxiety	DASS 21 items	-	Refined grain	7
37	Sangouni, 2022, [16] Iran	733	12–18	Depression	BDI	≥13	Refined grain/SSB/FFP	5
38	Hockey, 2023, [65] Finland	1600 (166)	63	Depression	DSM-III	≥5	HFD	6
39	Chatzi, 2011, [31] Greece	529 (176)	-	Depression	EPDS	≥13	HFD/Red meat	4

Abbreviations of diagnosis criteria: EPDS, Edinburg Postpartum Depression Scale; BDI, Beck Depression Inventory; BAI, Beck Anxiety Inventory; CES-D, Center for Epidemiologic Studies Depression Scale; K10, Kessler Psychological Distress Scale; PHQ-9, Patient Health Questionnaire 9-item depression module; HADS, Hospital Anxiety and Depression Scale; GHQ-12, General Health Questionnaire 12 items; SCID-I/NP, Structured Clinical Interview for DSM-IV-TR Research Version; CDI, The Children’s Depression Inventory; SASC, Social anxiety scale for children; SDS, the Chinese version of the Zung Self-Rating Depression Scale; GSHS, Global School Health Survey; DASS 21, depression, anxiety and stress scale; FEAR, Fear Questionnaire; GMS, the Geriatric Mental State; CONOR-MHI, the Conor Mental Health Index. Abbreviations of Dietary pattern type: HFD, high-fat dairy; SSB, sugar-sweetened beverage; FFP, fast food pattern.

**Table 3:** Subgroup analyses of western dietary pattern, red meat, and risk ratio of depression and depressive symptoms by pervious defined study characteristics.

Exposure and Outcomes	Factors	Variables	No. of Studies	RR (95% CI)	Test of Heterogeneity ^1^		*p* ^2^
					*p*	*I*^2^ (%)	
WDP and depressive symptoms	Gender	Female	4	1.06 (0.88–1.27)	0.06	59	0.53
		Male	3	1.36 (1.25–1.47)	0.99	0	<0.01
WDP and depression	Gender	Female	3	1.09 (0.95–1.27)	0.42	0	0.23
		Male	2	1.11 (0.98–1.24)	0.68	0	0.29
	Location	Western countries	3	1.08 (0.95–1.23)	0.46	0	0.21
		Eastern countries	4	1.48 (1.12–1.96)	0.15	43	<0.01
Red meat and depression	Number of participates	<2000	7	1.00 (0.99–1.02)	0.62	0	0.71
		2000–10,000	5	1.04 (0.97–1.12)	0.18	36	0.28
		>10,000	2	1.41 (1.24–1.61)	0.73	0	<0.01
	Location	Western countries	9	1.03 (0.98–1.07)	0.73	0	<0.01
		Eastern countries	6	1.41 (1.24–1.61)	0.73	0	<0.01

^1^ *p* for heterogeneity assessed by Cochran’s test, and *p* < 0.1 means significant heterogeneity across studies. The *I*^2^ calculated by Cochran’s test, and *I*^2^ > 50% means significant heterogeneity across studies. ^2^ *p* for meta-analysis: *p* < 0.01 means significant effect of exposure on the outcomes by using a random-effects model.

## Data Availability

The data presented in this study are available on request from the corresponding author.

## Supplementary Materials

The following supporting information can be downloaded at: https://www.mdpi.com/article/10.3390/nu16010125/s1, Supplementary Table S1. PRISMA Checklist for this systematic review and meta-analysis. Supplementary Table S2. MOOSE Checklist for this systematic review and meta-analysis. Supplementary Table S3. Quality assessment of all included studies. Supplementary Figure S1. Funnel plots of western dietary pattern and behavioral health disorders risk in the highest versus lowest analysis. Supplementary Figure S2. Funnel plots of fast food intake and behavioral health disorders risk in the highest versus lowest analysis. Supplementary Figure S3. Funnel plots of red meat intake and behavioral health disorders risk in the highest versus lowest analysis. Supplementary Figure S4. Funnel plots of refined grain intake and behavioral health disorders risk in the highest versus lowest analysis. Supplementary Figure S5. Funnel plots of sugar-sweeten beverage intake and behavioral health disorders risk in the highest versus lowest analysis. Supplementary Figure S6. Funnel plots of high-fat dairy intake and behavioral health disorders risk in the highest versus lowest analysis. Supplementary Figure S7. Subgroup analysis (stratified by different gender of participates) for western dietary pattern and risk of depression. Supplementary Figure S8. Subgroup analysis (stratified by different location of studies) for western dietary pattern and risk of depression. Supplementary Figure S9. Subgroup analysis (stratified by different gender of participates) for western dietary pattern and risk of depressive symptoms. Supplementary Figure S10. Subgroup analysis (stratified by different location of studies) for red meat intakes and risk of depression. Supplementary Figure S11. Subgroup analysis (stratified by number of participates) for red meat intakes and risk of depression.
