# Supplementary material for: Association between Western Dietary Patterns, Typical Food Groups, and Behavioral Health Disorders: An Updated Systematic Review and Meta-Analysis of Observational Studies
Source: Nutrients. 2023 Dec 29;16(1):125. doi: 10.3390/nu16010125 (PMC10780533; doi:10.3390/nu16010125)
Supplement: Supplementary file 1 [file nutrients-16-00125-s001.zip › nutrients-2714611-supplementary.pdf]

The association between western dietary patterns, typical food groups and behavioral health disorders: an updated systematic review and meta-analysis of observational studies

Wei Quan

Online Supplementary Material

CONTENTS:

Supplementary Table S1. PRISMA Checklist for this systematic review and meta-analysis

Supplementary Table S2. MOOSE Checklist for this systematic review and meta-analysis

Supplementary Table S3. Quality assessment of all included studies

Supplementary Figure S1. Funnel plots of western dietary pattern and behavioral health disorders risk in the highest *versus* lowest analysis.

Supplementary Figure S2. Funnel plots of fast food intake and behavioral health disorders risk in the highest *versus* lowest analysis.

Supplementary Figure S3. Funnel plots of red meat intake and behavioral health disorders risk in the highest *versus* lowest analysis.

Supplementary Figure S4. Funnel plots of refined grain intake and behavioral health disorders risk in the highest *versus* lowest analysis.

Supplementary Figure S5. Funnel plots of sugar-sweeten beverage intake and behavioral health disorders risk in the highest *versus* lowest analysis.

Supplementary Figure S6. Funnel plots of high-fat dairy intake and behavioral health disorders risk in the highest *versus* lowest analysis.

Supplementary Figure S7. Subgroup analysis (stratified by different gender of participates) for western dietary pattern and risk of depression.

Supplementary Figure S8. Subgroup analysis (stratified by different location of studies) for western dietary pattern and risk of depression.

Supplementary Figure S9. Subgroup analysis (stratified by different gender of participates) for western dietary pattern and risk of depressive symptoms.

Supplementary Figure S10. Subgroup analysis (stratified by different location of studies) for red meat intakes and risk of depression.

Supplementary Figure S11. Subgroup analysis (stratified by number of participates) for red meat intakes and risk of depression.

Supplementary Table S1. PRISMA Checklist for this systematic review and meta-analysis

Section and Topic	Item #	Checklist item	Location where item is reported
**TITLE**			
Title	1	Identify the report as a systematic review.	1
**ABSTRACT**			
Abstract	2	See the PRISMA 2020 for Abstracts checklist.	2
**INTRODUCTION**			
Rationale	3	Describe the rationale for the review in the context of existing knowledge.	3
Objectives	4	Provide an explicit statement of the objective(s) or question(s) the review addresses.	3
**METHODS**			
Eligibility criteria	5	Specify the inclusion and exclusion criteria for the review and how studies were grouped for the syntheses.	5, 6
Information sources	6	Specify all databases, registers, websites, organisations, reference lists and other sources searched or consulted to identify studies. Specify the date when each source was last searched or consulted.	5
Search strategy	7	Present the full search strategies for all databases, registers and websites, including any filters and limits used.	5
Selection process	8	Specify the methods used to decide whether a study met the inclusion criteria of the review, including how many reviewers screened each record and each report retrieved, whether they worked independently, and if applicable, details of automation tools used in the process.	5
Data collection	9	Specify the methods used to collect data from reports, including how many reviewers collected data from each report,	5, 6

Section and Topic	Item #	Checklist item	Location where item is reported
process		whether they worked independently, any processes for obtaining or confirming data from study investigators, and if applicable, details of automation tools used in the process.	
Data items	10a	List and define all outcomes for which data were sought. Specify whether all results that were compatible with each outcome domain in each study were sought (e.g. for all measures, time points, analyses), and if not, the methods used to decide which results to collect.	5, 6
	10b	List and define all other variables for which data were sought (e.g. participant and intervention characteristics, funding sources). Describe any assumptions made about any missing or unclear information.	5, 6
Study risk of bias assessment	11	Specify the methods used to assess risk of bias in the included studies, including details of the tool(s) used, how many reviewers assessed each study and whether they worked independently, and if applicable, details of automation tools used in the process.	6, 7
Effect measures	12	Specify for each outcome the effect measure(s) (e.g. risk ratio, mean difference) used in the synthesis or presentation of results.	6, 7
Synthesis methods	13a	Describe the processes used to decide which studies were eligible for each synthesis (e.g. tabulating the study intervention characteristics and comparing against the planned groups for each synthesis (item #5)).	6, 7
	13b	Describe any methods required to prepare the data for presentation or synthesis, such as handling of missing summary statistics, or data conversions.	6, 7
	13c	Describe any methods used to tabulate or visually display results of individual studies and syntheses.	6, 7
	13d	Describe any methods used to synthesize results and provide a rationale for the choice(s). If meta-analysis was performed, describe the model(s), method(s) to identify the presence and extent of statistical heterogeneity, and software package(s)	6, 7

Section and Topic	Item #	Checklist item	Location where item is reported
		used.	
	13e	Describe any methods used to explore possible causes of heterogeneity among study results (e.g. subgroup analysis, meta-regression).	6, 7
	13f	Describe any sensitivity analyses conducted to assess robustness of the synthesized results.	6, 7
Reporting bias assessment	14	Describe any methods used to assess risk of bias due to missing results in a synthesis (arising from reporting biases).	7
Certainty assessment	15	Describe any methods used to assess certainty (or confidence) in the body of evidence for an outcome.	7
**RESULTS**			
Study selection	16a	Describe the results of the search and selection process, from the number of records identified in the search to the number of studies included in the review, ideally using a flow diagram.	7
	16b	Cite studies that might appear to meet the inclusion criteria, but which were excluded, and explain why they were excluded.	7
Study characteristics	17	Cite each included study and present its characteristics.	7, 8
Risk of bias in studies	18	Present assessments of risk of bias for each included study.	10
Results of individual studies	19	For all outcomes, present, for each study: (a) summary statistics for each group (where appropriate) and (b) an effect estimate and its precision (e.g. confidence/credible interval), ideally using structured tables or plots.	8

Section and Topic	Item #	Checklist item	Location where item is reported
Results of syntheses	20a	For each synthesis, briefly summarise the characteristics and risk of bias among contributing studies.	8-10
	20b	Present results of all statistical syntheses conducted. If meta-analysis was done, present for each the summary estimate and its precision (e.g. confidence/credible interval) and measures of statistical heterogeneity. If comparing groups, describe the direction of the effect.	8-10
	20c	Present results of all investigations of possible causes of heterogeneity among study results.	8-10
	20d	Present results of all sensitivity analyses conducted to assess the robustness of the synthesized results.	8-10
Reporting biases	21	Present assessments of risk of bias due to missing results (arising from reporting biases) for each synthesis assessed.	10
Certainty of evidence	22	Present assessments of certainty (or confidence) in the body of evidence for each outcome assessed.	10
**DISCUSSION**			
Discussion	23a	Provide a general interpretation of the results in the context of other evidence.	12
	23b	Discuss any limitations of the evidence included in the review.	15, 16
	23c	Discuss any limitations of the review processes used.	15, 16
	23d	Discuss implications of the results for practice, policy, and future research.	15, 16
**OTHER INFORMATION**			
Registration and protocol	24a	Provide registration information for the review, including register name and registration number, or state that the review was not registered.	4

Section and Topic	Item #	Checklist item	Location where item is reported
	24b	Indicate where the review protocol can be accessed, or state that a protocol was not prepared.	4
	24c	Describe and explain any amendments to information provided at registration or in the protocol.	4
Support	25	Describe sources of financial or non-financial support for the review, and the role of the funders or sponsors in the review.	16
Competing interests	26	Declare any competing interests of review authors.	17
Availability of data, code and other materials	27	Report which of the following are publicly available and where they can be found: template data collection forms; data extracted from included studies; data used for all analyses; analytic code; any other materials used in the review.	17

Supplementary Table S2. MOOSE Checklist for this systematic review and meta-analysis

Criteria		Brief description of how the criteria were handled in the meta-analysis
**Reporting of background should include**		
√	Problem definition	An increasing number of studies have started to focus on the association between WDP and behavioral health
√	Hypothesis statement	the inflammatory potential of WDP in relation to behavioral disorders
√	Description of study outcomes	behavioral disorders
√	Type of exposure or intervention used	Western dietary pattern and food groups
√	Type of study designs used	We included observational studies
√	Study population	People without behavioral disorders
**Reporting of search strategy should include**		
√	Qualifications of searchers	The credentials of the two investigators are indicated in the author list.
√	Search strategy, including time period included in the synthesis and keywords	PubMed from 1990 – August 2023<br>EMBASE from 1990 –August 2023<br>MEDLINE 1990 –August 2023<br>Web of Knowledge 1990 –August 2023<br>The Cochrane Library 1990 –August 2023<br>Keywords See search strategy section in the article
√	Databases and registries searched	PubMed, Embase, MEDLINE, Web of Knowledge, and the Cochrane Library
√	Search software used, name and version, including special features	We did not employ a search software. EndNote was used to merge retrieved citations and eliminate duplications
√	Use of hand searching	We have hand-checked the reference lists of original publications and previous meta-analyses or reviews
√	List of citations located and those excluded, including justifications	Details of the literature search process are outlined in the flow chart. The citation list is available upon request
√	Method of addressing articles published in languages other than English	We limited to studies published in the English language
√	Method of handling abstracts and unpublished studies	Unpublished data, conference papers, editorials, theses, and patents were not included
√	Description of any contact with authors	We contacted corresponding authors of studies that did not reported sufficient data in an effort to complete our data set.
**Reporting of methods should include**		
√	Description of relevance or appropriateness of studies assembled for assessing the hypothesis to be tested	Detailed inclusion and exclusion criteria were described in the methods section.
√	Rationale for the selection and coding of data	Data extracted from each of the studies were relevant to the first author's name; year of publication; country; duration of follow-up; age range; number of participants and incident cases; diagnostic method and criteria of outcome; dietary assessment method; food items; multivariate-adjusted risk estimate; and confounding factors of interest
√	Assessment of confounding	Restricted the analysis to meat estimates only.
√	Assessment of study quality, including blinding of quality assessors; stratification or regression on possible predictors of study results	The Newcastle–Ottawa Scale (NOS) adapted for cohort studies was used by two investigators to assess the quality of the included articles.

√	Assessment of heterogeneity	Heterogeneity of the studies were explored within two types of study designs using Cochrane's Q test of heterogeneity and I^2 statistic that provides the relative amount of variance of the summary effect due to the between-study heterogeneity.
√	Description of statistical methods in sufficient detail to be replicated	Description of methods of meta-analyses, sensitivity analyses, subgroup analysis and assessment of publication bias are detailed in the methods.
√	Provision of appropriate tables and graphics	We included 1 flow chart,1 summary table, 1table of subgroup analysis, 6 forest plot of all studies, 1 table of sensitivity analyses.
**Reporting of results should include**		
√	Graph summarizing individual study estimates and overall estimate	Figure 1
√	Table giving descriptive information for each study included	Table 1
√	Results of sensitivity testing	Supplementary Table 1
√	Indication of statistical uncertainty of findings	95% confidence intervals were presented with all summary estimates, I^2 values and results of sensitivity analyses
**Reporting of discussion should include**		
√	Quantitative assessment of bias	Sensitivity analyses indicate heterogeneity in strengths of the association due to most common biases in observational studies.
√	Justification for exclusion	We excluded studies that had not reported for the meat consumption as exposure
√	Assessment of quality of included studies	We discussed the results of the sensitivity analyses, and potential reasons for the observed heterogeneity.
**Reporting of conclusions should include**		
√	Consideration of alternative explanations for observed results	We discussed that potential unmeasured confounders such as Maillard reaction harmful products may related to the risk of cognitive disorders.
√	Generalization of the conclusions	A high consumption of total meat (especially for processed total meat and red meat) is associated with increased risk of neurodegenerative cognitive disorders. While higher fish and poultry intakes is associated with decreased risk of neurodegenerative cognitive disorders
√	Guidelines for future research	We recommend to exploring the potential mechanisms of processed meat products or their harmful products and the risk of cognitive impairment in the future.
√	Disclosure of funding source	This work has been supported by the National Natural Science Foundation of China (Grant No. 3217160166)

Supplementary Table S3 Quality assessment of the publications included in the meta-analysis

No.	selection of the study groups 0-4	Adjustment for known confounding factors 0-2	Ascertainment of the outcome of interest 0-3	Total score	No.	selection of the study groups 0-4	Adjustment for known confounding factors 0-2	Ascertainment of the outcome of interest 0-3	Total score
1	2	1	2	5	21	3	1	2	6
2	2	2	2	6	22	3	2	2	7
3	2	2	2	6	23	3	2	2	7
4	4	2	2	8	24	2	2	1	5
5	2	2	2	6	25	2	1	2	5
6	2	2	1	5	26	3	2	2	7
7	2	2	2	6	27	3	2	2	7
8	2	1	2	5	28	2	1	2	5
9	2	2	1	5	29	1	2	2	5
10	2	2	2	6	30	2	2	2	6
11	3	2	2	7	31	2	2	1	5
12	2	1	1	4	32	3	2	2	7
13	1	1	1	4	33	2	2	2	6
14	2	2	2	6	34	3	2	2	7
15	2	2	1	5	35	3	1	2	6
16	2	2	1	5	36	3	1	3	7
17	1	1	2	4	37	2	1	2	5
18	3	2	2	7	38	3	2	2	7
19	2	2	2	6	39	2	2	2	6
20	1	2	2	5	40	2	2	2	6
41	3	1	2	6	48	2	1	2	5
42	1	2	2	5	49	2	1	2	5
43	2	2	1	5	50	3	1	1	5
44	2	2	2	6	51	2	2	1	5
45	1	2	2	5	52	3	2	2	7
46	2	2	1	5	53	2	2	1	5
47	3	2	2	7	54	2	2	2	6

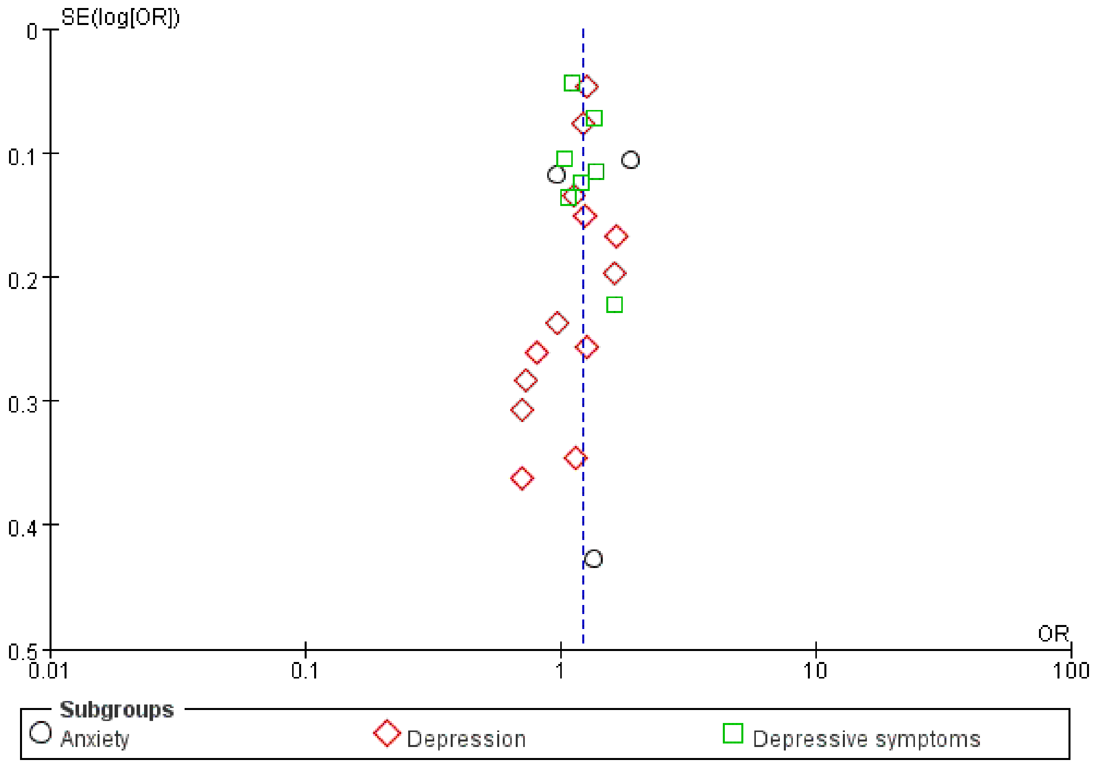

Supplementary Figure S1. Funnel plots of western dietary pattern and behavioral health disorders risk in the highest *versus* lowest analysis.

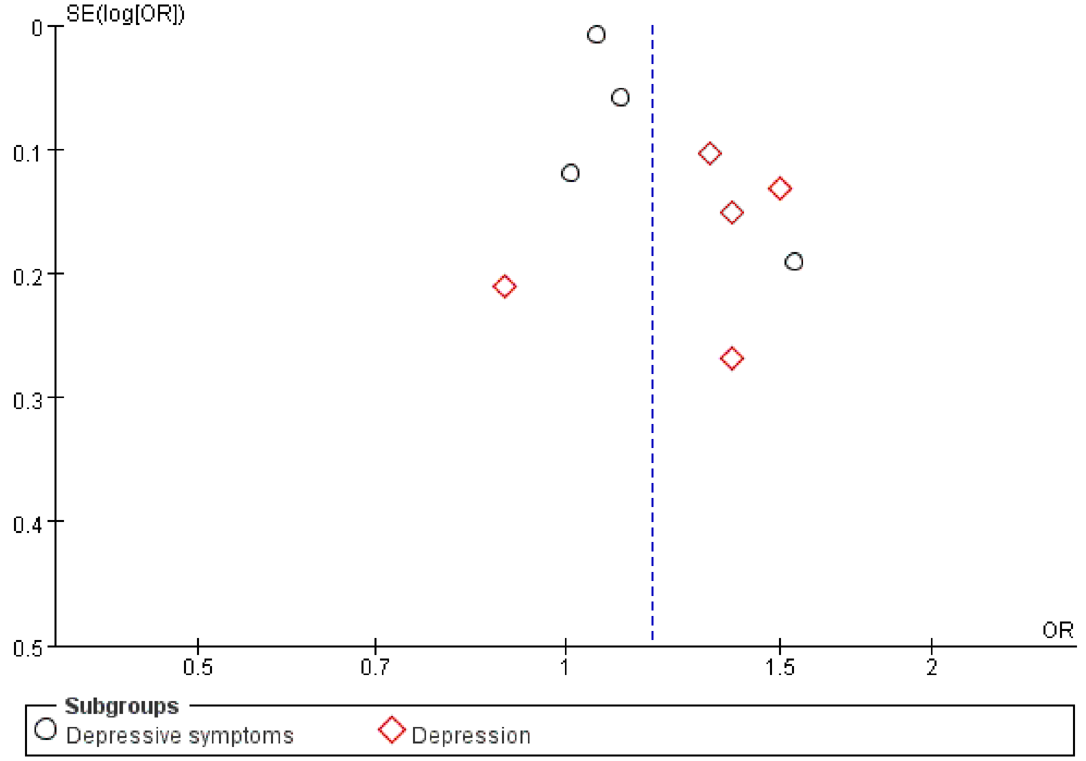

Supplementary Figure S2. Funnel plots of fast food intake and behavioral health disorders risk in the highest *versus* lowest analysis.

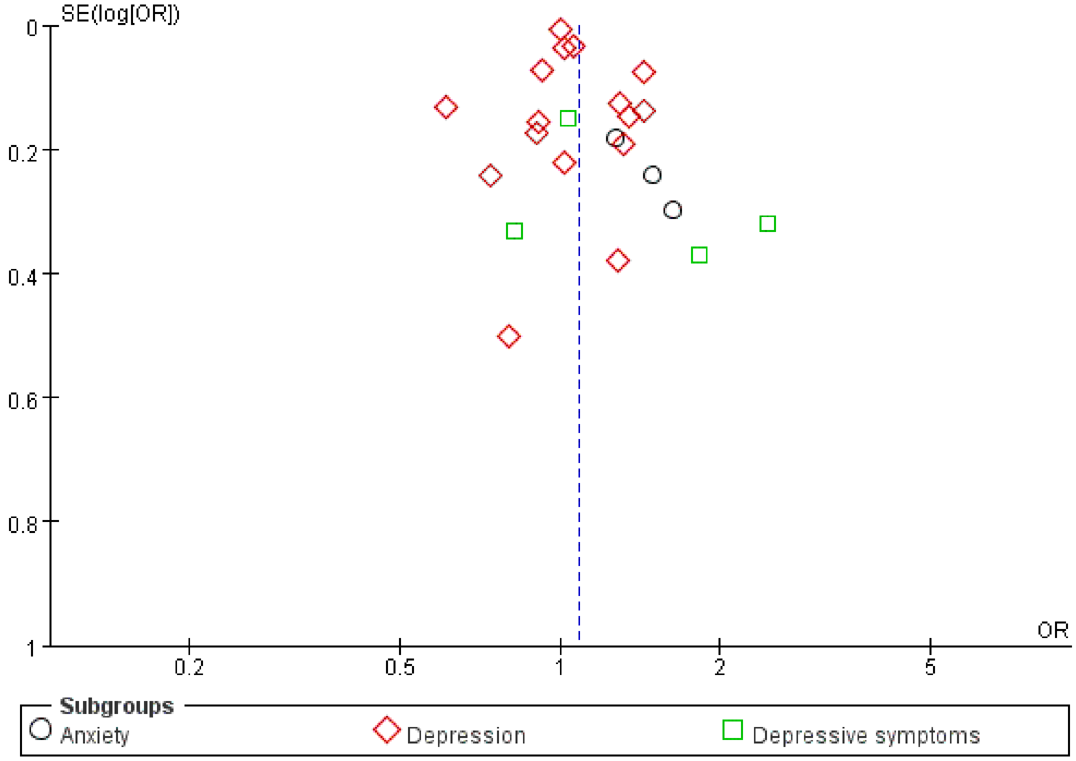

Supplementary Figure S3. Funnel plots of red meat intake and behavioral health disorders risk in the highest *versus* lowest analysis.

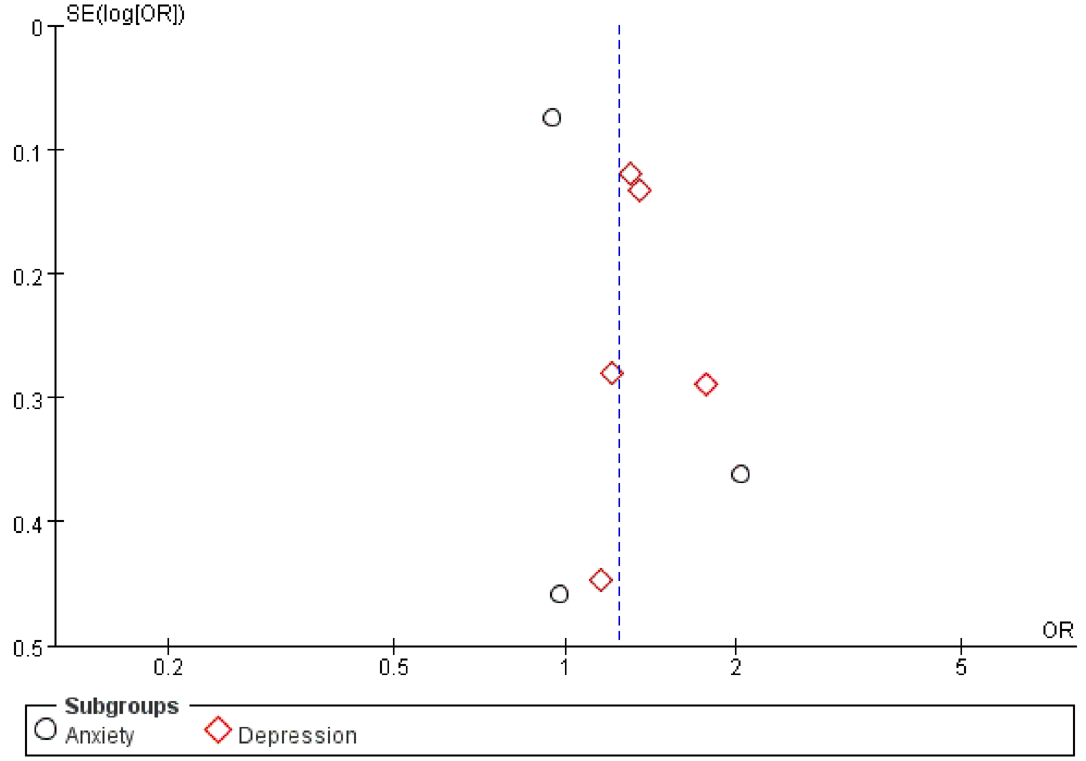

Supplementary Figure S4. Funnel plots of refined grain intake and behavioral health disorders risk in the highest *versus* lowest analysis.

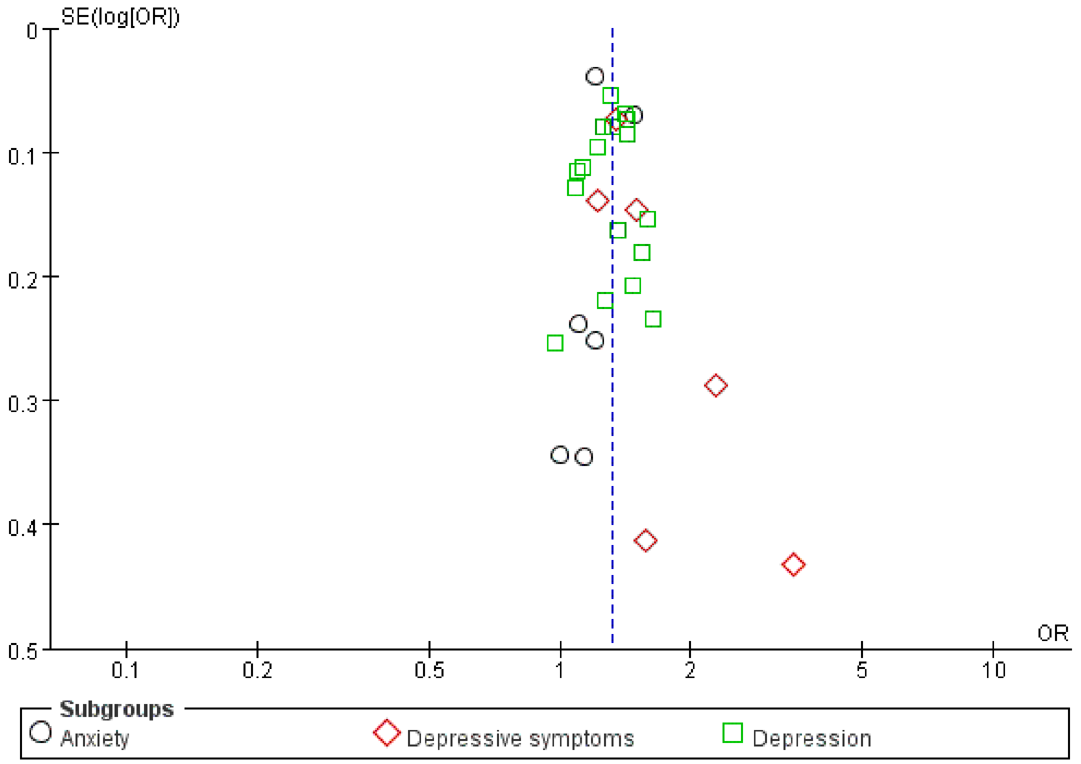

Supplementary Figure S5. Funnel plots of sugar-sweeten beverage intake and behavioral health disorders risk in the highest *versus* lowest analysis.

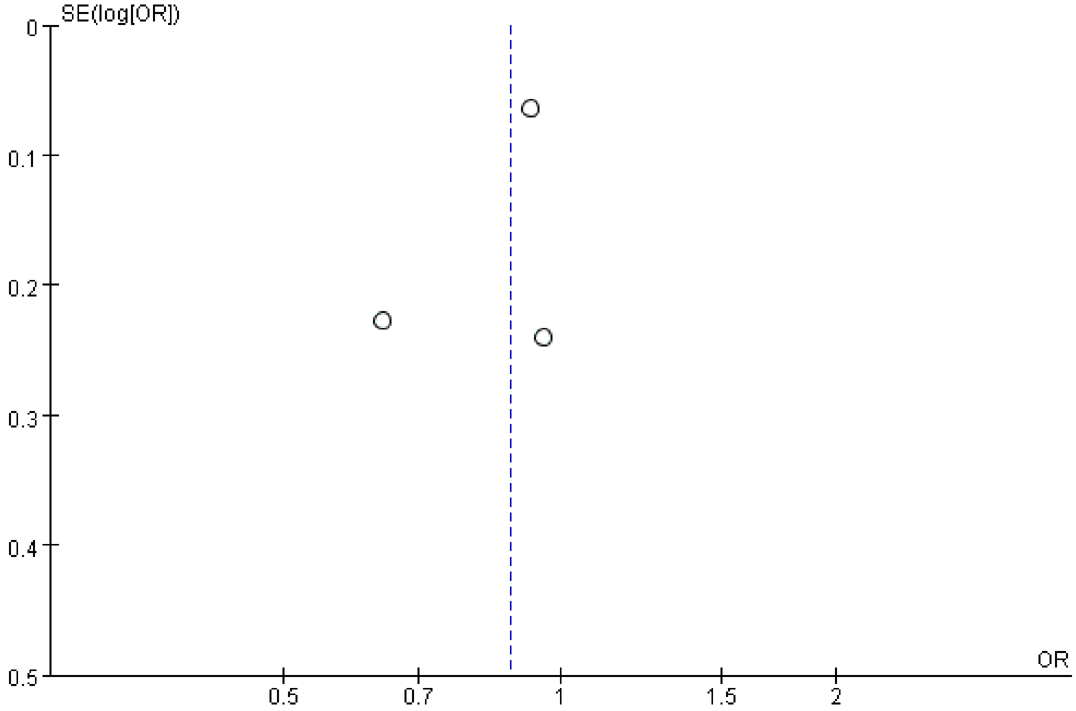

Supplementary Figure S6. Funnel plots of high-fat dairy intake and behavioral health disorders risk in the highest *versus* lowest analysis.

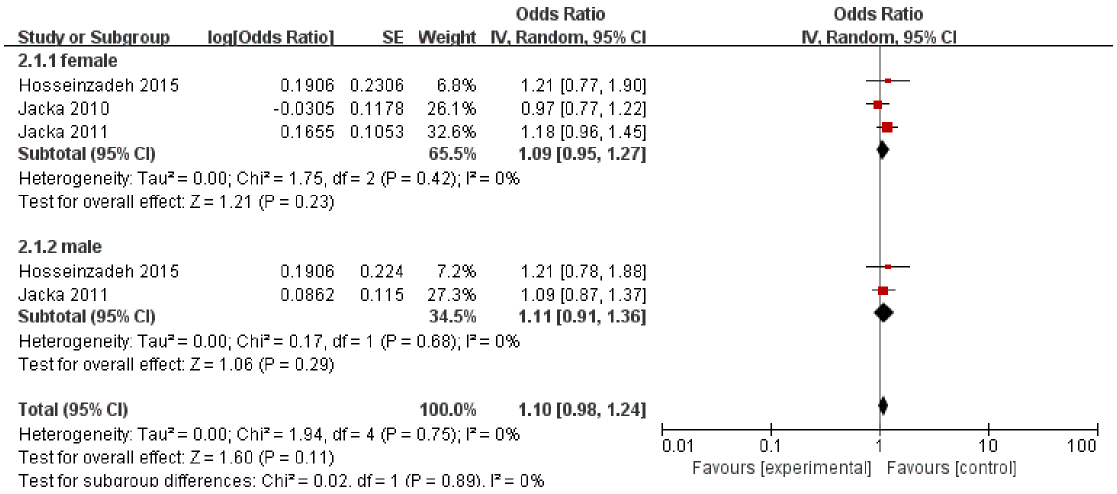

Supplementary Figure S7. Subgroup analysis (stratified by different gender of participates) for western dietary pattern and risk of depression.

Study or Subgroup	log[Odds Ratio]	SE	Weight	Odds Ratio IV, Random, 95% CI
**2.2.1 western**				
Jacka 2010	-0.0305	0.1178	17.7%	0.97 [0.77, 1.22]
Jacka 2011	0.1655	0.1053	18.5%	1.18 [0.96, 1.45]
Jacka 2011	0.0862	0.115	17.9%	1.09 [0.87, 1.37]
**Subtotal (95% CI)**			**54.2%**	**1.08 [0.95, 1.23]**
Heterogeneity: Tau² = 0.00; Chi² = 1.54, df = 2 (P = 0.46); I² = 0%				
Test for overall effect: Z = 1.25 (P = 0.21)				
**2.2.2 Eastern**				
Hosseinzadeh 2015	0.3075	0.4261	4.9%	1.36 [0.59, 3.14]
Hosseinzadeh 2015	0.1906	0.224	11.3%	1.21 [0.78, 1.88]
Hosseinzadeh 2015	0.1906	0.2306	11.0%	1.21 [0.77, 1.90]
Weng 2011	0.6259	0.1057	18.5%	1.87 [1.52, 2.30]
**Subtotal (95% CI)**			**45.8%**	**1.48 [1.12, 1.96]**
Heterogeneity: Tau² = 0.03; Chi² = 5.31, df = 3 (P = 0.15); I² = 43%				
Test for overall effect: Z = 2.79 (P = 0.005)				
**Total (95% CI)**			**100.0%**	**1.24 [1.01, 1.53]**
Heterogeneity: Tau² = 0.05; Chi² = 20.95, df = 6 (P = 0.002); I² = 71%				
Test for overall effect: Z = 2.01 (P = 0.04)				
Test for subgroup differences: Chi² = 4.06, df = 1 (P = 0.04), I² = 75.3%				

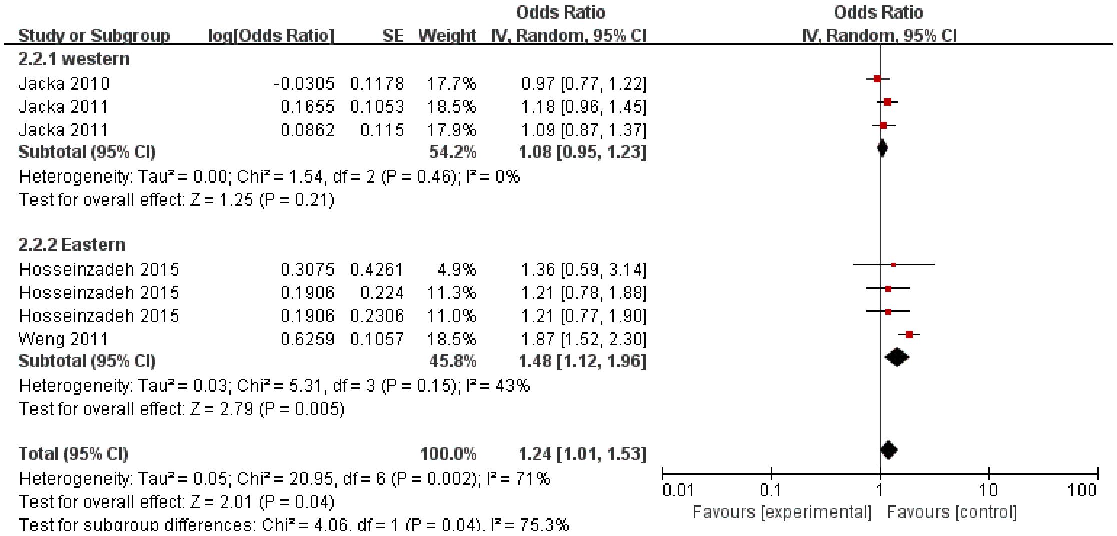

Supplementary Figure S8. Subgroup analysis (stratified by different location of studies) for western dietary pattern and risk of depression.

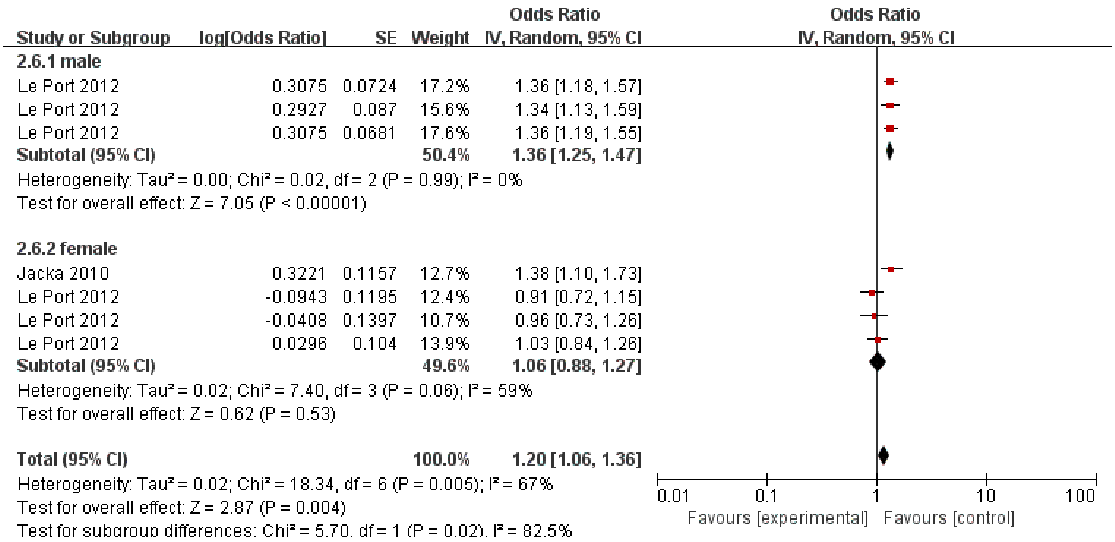

Supplementary Figure S9. Subgroup analysis (stratified by different gender of participates) for western dietary pattern and risk of depressive symptoms.

Study or Subgroup	log[Odds Ratio]	SE	Weight	Odds Ratio IV, Random, 95% CI
**2.3.1 western**				
Chatzi 2011	0.2469	0.3781	0.8%	1.28 [0.61, 2.69]
Gibson-Smith 2020	-0.0834	0.0713	10.9%	0.92 [0.80, 1.06]
Lampignano 2022	0.003	0.0067	20.3%	1.00 [0.99, 1.02]
Moludi 2020	0.2546	0.1248	5.6%	1.29 [1.01, 1.65]
Rienks 2013	0.0583	0.0349	16.9%	1.06 [0.99, 1.14]
Rienks 2013	0.0198	0.0363	16.7%	1.02 [0.95, 1.10]
Sanchez-Villegas 2009	0.3001	0.148	4.3%	1.35 [1.01, 1.80]
Wolfe 2011	0.0198	0.2221	2.2%	1.02 [0.66, 1.58]
Wolfe 2011	-0.0943	0.1562	4.0%	0.91 [0.67, 1.24]
**Subtotal (95% CI)**			**81.7%**	**1.03 [0.98, 1.07]**
Heterogeneity: Tau² = 0.00; Chi² = 12.90, df = 8 (P = 0.12); I² = 38%				
Test for overall effect: Z = 1.08 (P = 0.28)				
**2.3.2 eastern**				
Chen 2005	-0.2231	0.5004	0.5%	0.80 [0.30, 2.13]
Kazemi 2021	0.3577	0.1385	4.8%	1.43 [1.09, 1.88]
Miyake 2013	-0.1054	0.1739	3.3%	0.90 [0.64, 1.27]
Sangsefidi 2020	-0.3011	0.2426	1.8%	0.74 [0.46, 1.19]
Tsai 2011	0.27	0.1915	2.8%	1.31 [0.90, 1.91]
Zhou 2014	-0.4943	0.133	5.1%	0.61 [0.47, 0.79]
**Subtotal (95% CI)**			**18.3%**	**0.94 [0.67, 1.31]**
Heterogeneity: Tau² = 0.13; Chi² = 23.89, df = 5 (P = 0.0002); I² = 79%				
Test for overall effect: Z = 0.38 (P = 0.71)				
**Total (95% CI)**			**100.0%**	**1.02 [0.95, 1.09]**
Heterogeneity: Tau² = 0.01; Chi² = 37.61, df = 14 (P = 0.0006); I² = 63%				
Test for overall effect: Z = 0.58 (P = 0.56)				
Test for subgroup differences: Chi² = 0.27, df = 1 (P = 0.60), I² = 0%				

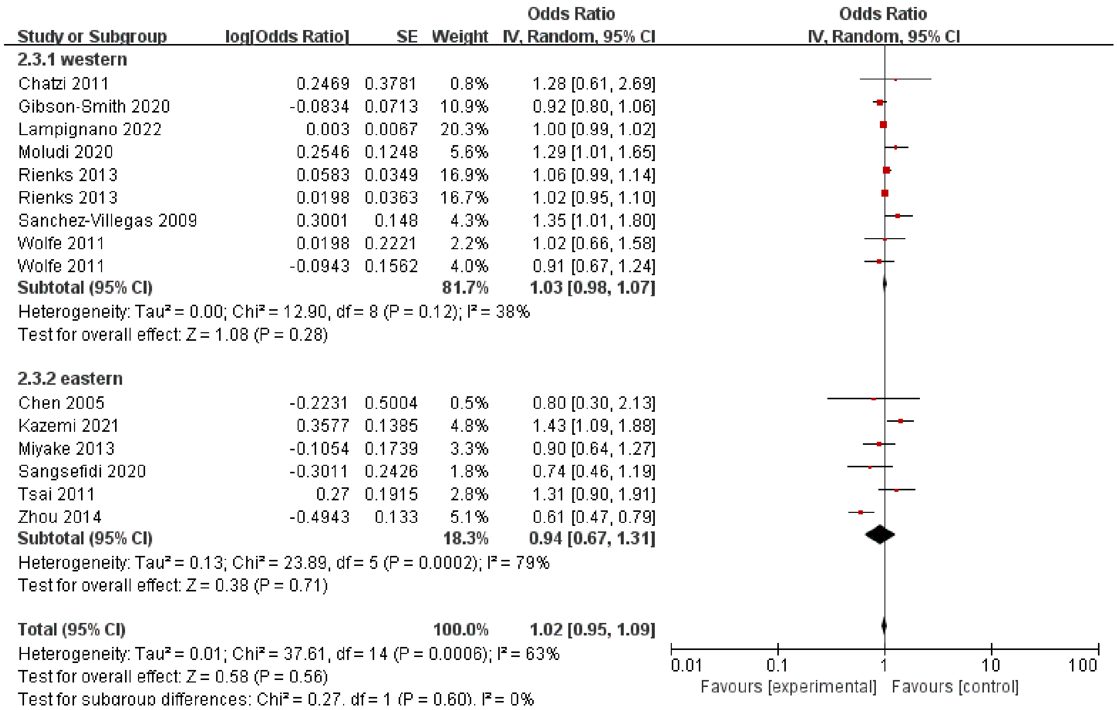

Supplementary Figure S10. Subgroup analysis (stratified by different location of studies) for red meat intakes and risk of depression.

Study or Subgroup	log[Odds Ratio]	SE	Weight	Odds Ratio IV, Random, 95% CI
**2.5.1 <2000**				
Chatzi 2011	0.2469	0.3781	0.8%	1.28 [0.61, 2.69]
Chen 2005	-0.0834	0.0713	10.9%	0.92 [0.80, 1.06]
Gibson-Smith 2020	0.27	0.1915	2.8%	1.31 [0.90, 1.91]
Lampignano 2022	0.0198	0.2221	2.2%	1.02 [0.66, 1.58]
Miyake 2013	-0.2231	0.5004	0.5%	0.80 [0.30, 2.13]
Tsai 2011	-0.1054	0.1739	3.3%	0.90 [0.64, 1.27]
Wolfe 2011	0.003	0.0067	20.1%	1.00 [0.99, 1.02]
**Subtotal (95% CI)**			**40.7%**	**1.00 [0.99, 1.02]**
Heterogeneity: Tau² = 0.00; Chi² = 4.42, df = 6 (P = 0.62); I² = 0%				
Test for overall effect: Z = 0.37 (P = 0.71)				
**2.5.2 2000-10000**				
Moludi 2020	-0.3011	0.2426	1.9%	0.74 [0.46, 1.19]
Rienks 2013	0.0583	0.0349	16.8%	1.06 [0.99, 1.14]
Rienks 2013	0.0198	0.0363	16.6%	1.02 [0.95, 1.10]
Sangsefidi 2020	-0.0943	0.1562	4.0%	0.91 [0.67, 1.24]
Wolfe 2011	0.2546	0.1248	5.6%	1.29 [1.01, 1.65]
**Subtotal (95% CI)**			**44.8%**	**1.04 [0.97, 1.12]**
Heterogeneity: Tau² = 0.00; Chi² = 6.25, df = 4 (P = 0.18); I² = 36%				
Test for overall effect: Z = 1.07 (P = 0.28)				
**2.5.3 >10000**				
Sanchez-Villegas 2009	0.3001	0.148	4.3%	1.35 [1.01, 1.80]
Zhou 2014	0.3577	0.0769	10.2%	1.43 [1.23, 1.66]
**Subtotal (95% CI)**			**14.5%**	**1.41 [1.24, 1.61]**
Heterogeneity: Tau² = 0.00; Chi² = 0.12, df = 1 (P = 0.73); I² = 0%				
Test for overall effect: Z = 5.06 (P < 0.00001)				
**Total (95% CI)**			**100.0%**	**1.07 [1.00, 1.14]**
Heterogeneity: Tau² = 0.01; Chi² = 37.83, df = 13 (P = 0.0003); I² = 66%				
Test for overall effect: Z = 1.85 (P = 0.06)				
Test for subgroup differences: Chi² = 25.85, df = 2 (P < 0.00001), I² = 92.3%				

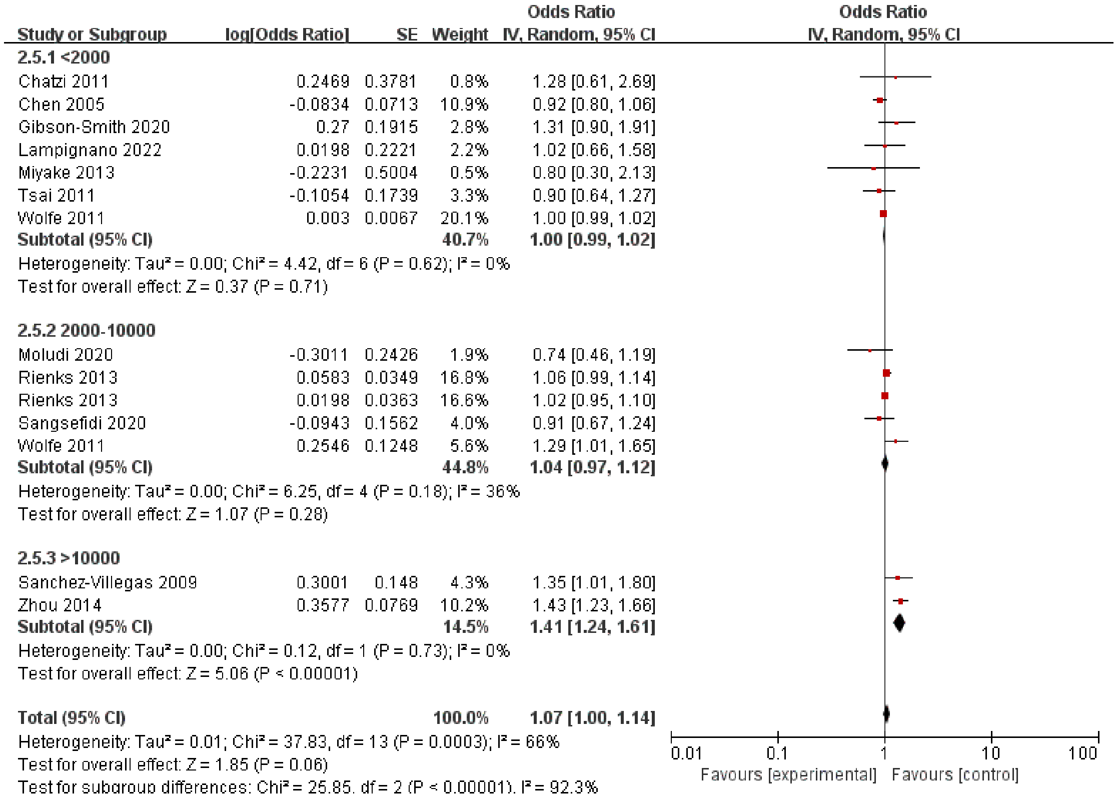

Supplementary Figure S11. Subgroup analysis (stratified by number of participates) for red meat intakes and risk of depression.
